# Heterogeneity of surrogate outcome measures used in critical care studies: A systematic review

**DOI:** 10.1177/17407745231151842

**Published:** 2023-03-22

**Authors:** Rejina Verghis, Bronagh Blackwood, Cliona McDowell, Philip Toner, Daniel Hadfield, Anthony C Gordon, Mike Clarke, Daniel McAuley

**Affiliations:** 1 The Wellcome-Wolfson Institute for Experimental Medicine, School of Medicine, Dentistry and Biomedical Sciences, Queen’s University Belfast, Belfast, UK; 2 Northern Ireland Clinical Trials Unit, Belfast, UK; 3 Critical Care Unit, King’s College Hospital NHS Foundation Trust, London, UK; 4 Division of Anaesthetics, Pain Medicine and Intensive Care, Imperial College London, London, UK; 5 Centre of Public Health, School of Medicine, Dentistry and Biomedical Sciences, Queen’s University Belfast, Belfast, UK

**Keywords:** Clinical trials, critical care, outcomes

## Abstract

**Background:**

The choice of outcome measure is a critical decision in the design of any clinical trial, but many Phase III clinical trials in critical care fail to detect a difference between the interventions being compared. This may be because the surrogate outcomes used to show beneficial effects in early phase trials (which informed the design of the subsequent Phase III trials) are not valid guides to the differences between the interventions for the main outcomes of the Phase III trials. We undertook a systematic review (1) to generate a list of outcome measures used in critical care trials, (2) to determine the variability in the outcome reporting in the respiratory subgroup and (3) to create a smaller list of potential early phase endpoints in the respiratory subgroup.

**Methods:**

Data related to outcomes were extracted from studies published in the six top-ranked critical care journals between 2010 and 2020. Outcomes were classified into subcategories and categories. A subset of early phase endpoints relevant to the respiratory subgroup was selected for further investigation. The variability of the outcomes and the variability in reporting was investigated.

**Results:**

A total of 6905 references were retrieved and a total of 294 separate outcomes were identified from 58 studies. The outcomes were then classified into 11 categories and 66 subcategories. A subset of 22 outcomes relevant for the respiratory group were identified as potential early phase outcomes. The summary statistics, time points and definitions show the outcomes are analysed and reported in different ways.

**Conclusion:**

The outcome measures were defined, analysed and reported in a variety of ways. This creates difficulties for synthesising data in systematic reviews and planning definitive trials. This review once again highlights an urgent need for standardisation and validation of surrogate outcomes reported in critical care trials. Future work should aim to validate and develop a core outcome set for surrogate outcomes in critical care trials.

## Introduction

A clinical endpoint can be defined as a characteristic or variable that reflects on how a patient feels or functions, and how long a patient survives, whereas a surrogate endpoint is a marker intended to substitute for a clinical endpoint that should predict clinical benefit or harm or lack of both.^1^ It is often assumed that the surrogate outcomes mimic the clinical endpoint. The validity of a surrogate measure is often hard to prove. One reason for this may be that it is never tested and only a few datasets containing both the surrogate outcome measures and the corresponding clinical endpoint are available for use.

The use of non-validated outcome measures to inform intervention studies, especially confirmatory studies, can cause serious issues. A classic example of this was the Cardiac Arrhythmia Suppression Trial (CAST) I study in 1989 and CAST II in 1992, where the intervention effectively suppressed ventricular arrhythmias (surrogate) but was later shown to increased arrhythmic associated deaths (clinical endpoint).^2–4^ In an intensive care unit (ICU)-specific example, the double-blind study of the nitric oxide synthase inhibitor 546C88 showed that the intervention arm significantly improved shock resolution at 72 h (surrogate) in patients with severe sepsis, yet increased the mortality rate (clinical endpoint).^5–7^

The choice of outcome measure can vary based on the study design and objective. Phase II and III studies have different objectives; Phase II studies aim to provide an initial estimate of the effect size to inform the sample size of a Phase III study and are thus intended to inform the Phase III studies. In Phase II studies, investigators often use a surrogate or a short-term outcome measure to provide an initial estimate of intervention efficacy, whereas Phase III studies more often use a clinical endpoint. For example, the change in Sequential Organ Failure Assessment (SOFA) score, which is a composite of daily physiological measurements, can be considered as an outcome measure for a Phase II study, whereas a Phase III study would use a clinical endpoint such as 60-day mortality or long-term quality of life as the efficacy outcome measure. However, it is not always evident how the effect estimates of Phase II studies, based on surrogate outcome measures, inform the design of Phase III studies, which would use a different outcome measure. For example, how might an effect size based on the change in SOFA score at day 3 reflect changes in 60-day mortality? Many Phase III critical care studies fail to detect a difference between the groups, possibly because the surrogate outcome measures used in early phase studies may not be valid. This is a significant issue as the sample size and resource requirements for Phase III studies are much larger compared with a Phase II study.

Variability of the outcome measures and their definitions is another issue that leads to inconsistencies in research findings and their application. Variability in the outcome measures reported for randomised controlled trials (RCTs) makes any meaningful comparisons between studies difficult. Several authors have previously called for standardisation of outcomes definitions in critical care studies.^8–10^ In 1979, the World Health Organization (WHO) published a handbook on results reporting in cancer studies, which is considered the earliest attempt to standardise outcome reporting in healthcare research.^11^ The Outcome Measures in Rheumatoid Arthritis Clinical Trials Group (OMERACT) was the first group to formally recognise the variability in outcome selection and reporting in Rheumatology studies. The OMERACT network was initiated in 1992 and consensus conferences run biennially.^12^ The recommendations are data-driven and are updated by relevant working groups. The OMERACT model was followed by other medical fields including critical care. The Core Outcome Measures in Effectiveness Trial (COMET) initiative launched in 2010 leads the creation of Core Outcome Sets to improve standardisation and reporting in effectiveness studies.^9^ The International Forum for Acute Care Trialists (InFACT) collaboration established in 1989 drives outcomes research in critical care studies.^13^ The National Heart, Lung and Blood Institute and the Society of Critical Care Medicine had highlighted a need to gain consensus on a standard set of long-term outcomes for post-ICU discharge studies.^14^

The aim of this systematic review was to determine different outcomes reported in critical care trials, to determine the variability in outcome reporting in the respiratory subgroup, and to create a smaller list of potential early phase endpoints for critical care trials in the respiratory subgroup.

## Methods

This section details the systematic review methodology and the details of data extraction and data synthesis.

### Types of studies

Critical care RCTs involving adult patients were included in this review. All design types were included. Studies published in six journals in the critical care category with the highest impact factor at the time were included in this review. This restriction was made on the assumption that the studies would provide a good representation of outcomes reported in critical care trials. All interventions, pharmacological, non-pharmacological and medical device were included in this review, based on the assumption that the studies aimed to improve the clinical and efficacy outcomes of the patients and these outcomes were similar across several types of interventions.

### Type of participants

Studies were included if the participants were adult patients in intensive care units. Healthy volunteer studies, paediatric studies, end of life studies and transplant studies were excluded.

### Search methods

Studies were those published in the *American Journal of Respiratory and Critical Care Medicine, Chest, Critical Care, Critical Care Medicine, Intensive Care Medicine* and *Lancet Respiratory Medicine*, between the years 2010 and 2020. The search used MeSH headings, keywords and variants for ‘intensive care unit’ or ‘critical care’ combined (using the Boolean operator AND) with search strings to identify RCTs. The review was conducted according to the protocol published on the PROSPERO website (http://www.crd.york.ac.uk/PROSPERO/display_record.asp?ID=CRD42015017607).^15^

Studies investigating interventions for Corona Virus Disease 2019 (COVID-19) were not included in this review. A separate search was conducted on https://clinicaltrials.gov/ database, on Jan 2022, to identify the outcomes as per the trial registration for COVID-19 studies. Search terms used were COVID-19, COVID and corona virus. The outcomes identified in this review were compared with COVID study outcomes.

### Study selection

One reviewer conducted the initial search and screening. The study title, author names, abstract and journal names were extracted. Two reviewers examined the title and abstract of studies identified by the search. Full text of the studies deemed potentially suitable were retrieved and read to confirm eligibility. All studies published in 2010 which reported on surrogate outcome measures were considered for data extraction, and 10% of studies from 2014 and 2015. A further search was conducted in July 2020 and 10% of articles published in the past year were also extracted to see whether there was any significant change in the outcomes reported in these more recent years. The eligible articles were listed in an Excel sheet and 10% of the articles were randomly selected from the list.

### Data extraction

Details on all outcomes reported by the treatment arm were extracted. Baseline characteristics were not extracted. Details on the trial registration, outcome measures, definitions, time points and statistics were extracted.

The specific measurement variable, which corresponds to the data collected directly from trial participants (e.g. SOFA score); the participant-level analysis metric, which corresponds to the format of the outcome data that will be used from each trial participant for analysis (e.g. change from baseline, time to event); the method of aggregation, which refers to the summary measure format for each study group (e.g. mean, proportion); and the specific measurement time point of interest for analysis were extracted.

### Data cleaning and analysis

All outcomes except mortality and quality of life outcomes were considered as a surrogate outcome for this review. Outcomes related to safety and compliance were excluded since safety and compliance were not considered as outcomes that would give an initial estimate of the drug efficacy. Similarly, outcomes that were not specified in the methods section of the article were also excluded. If a study reported on SOFA score at days 1, 3 and 7, it was counted as one outcome. If a study reported the absolute SOFA score and change in SOFA (e.g. from baseline to day 7), this was counted as two outcomes.

At first, outcomes were arbitrarily categorised into body organ systems, biomarkers, disease severity score and resource use, and then into subcategories. Composite outcomes were placed into one category based on the most relevant component in the outcome. For example, ventilator free days which consist of components mortality and mechanical ventilation were classified under mechanical ventilation, because mortality outcomes were not included in this review and mechanical ventilation was the most relevant component. A smaller list of outcomes relevant to the respiratory subgroup was then considered for further analysis. The clinical relevance of the outcome was determined by one of the authors who is an ICU clinician and a leading critical care researcher. All data were entered into Microsoft Excel for analysis. Outcomes were tabulated to understand the patterns and variability. Pivot charts, pivot tables and sun charts were used to report the results. Counts and percentages were used to summarise the results.

## Results

**Figure 1.**
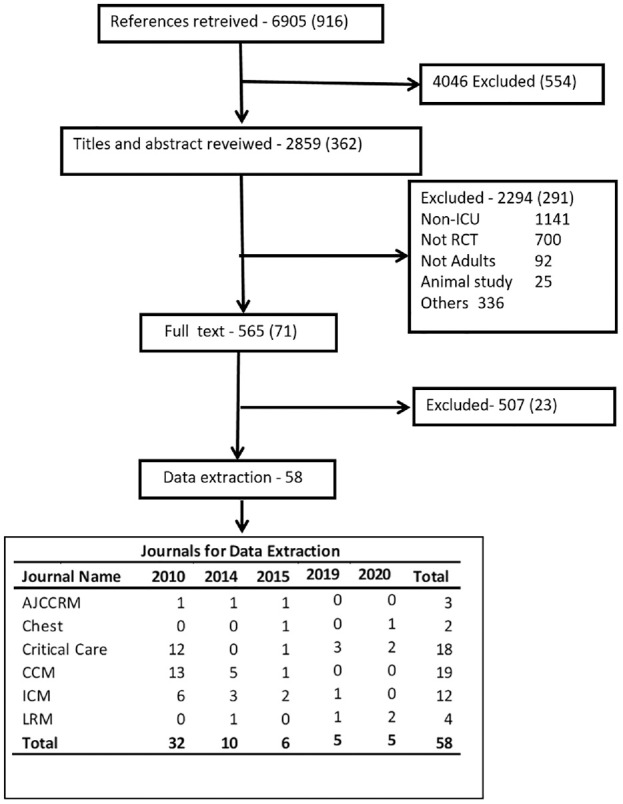
Study flowchart. AJRCCM: *American Journal of Respiratory and Critical Care Medicine*; CCM: *Critical Care Medicine*; ICM: *Intensive Care Medicine*; LRM: *Lancet Respiratory Medicine*. Numbers for 2010 are given in brackets.

A total of 6905 references were retrieved and 4046 were excluded at the initial screening as the inclusion criteria were not met. Titles and abstracts of 2859 studies were reviewed. Full text of 465 studies was retrieved. Data were extracted from 58 studies.^16–74^ The flow chart in Figure 1 shows the study selection process.

### Study characteristics

Studies included pharmacological 33 (56.9%), non-pharmacological 16 (27.6%) and medical device 9 (15.5%) interventions. Table 1 summarises the intervention type and patient condition. Nineteen (32.8%) were infection-related studies and 16 (27.6%) involved respiratory illness.

**Table 1.** Study characteristics.

	N
Total number of articles	58
Intervention	
Pharmacological	33 (56.9%)
Non-pharmacological	16 (27.6%)
Medical device	9 (15.5%)
Condition	
Infections and infestations	19 (32.8%)
Respiratory, thoracic, and mediastinal disorders	16 (27.6%)
Nervous system disorders	9 (15.5%)
General critical illness	4 (6.9%)
Cardiac disorders	3 (5.2%)
Renal and urinary disorders	2 (3.4%)
Metabolism and nutrition disorders	2 (3.4%)
Gastrointestinal disorders	2 (3.4%)
Surgical and medical procedures	1 (1.7%)

### Outcome categories and subcategories

The outcome measures, analysis metrics, time points and aggregation methods from the studies were identifiable for most of the outcomes. However, these parameters varied from trial to trial. Outcome measures were re-extracted for five studies by the second reviewer. There was 100% agreement after resolving the differences.

A total of 294 separate outcomes were identified from 58 studies. The 294 outcome measures were grouped into 11 categories and 66 subcategories; 33 (57%) studies reported on resource use outcome and 30 (52%) studies reported on cardiovascular outcomes. There were 50 (17%) cardiovascular outcomes, 41 (18%) respiratory outcomes and 46 (16%) infection outcomes. Table 2 shows the outcome categories, subcategories, number of outcomes identified and number of studies reporting the outcomes. Appendix C in the supplementary material elaborates on the outcomes and categories in Table 2.

**Table 2.** Summary table on outcome classification.

Category	Number of subcategories identified	Number of outcomes identified	Number of studies reporting the outcomes
Biomarker	2	20	14
Blood and lymphatic system	2	16	10
Cardiovascular system	6	50	30
Hepatobiliary system	1	5	8
Infection	13	46	22
Metabolism and nutrition system	6	18	10
Nervous system	12	33	13
Renal and urinary system	4	19	21
Resource	5	12	33
Respiratory, thoracic and mediastinal system	11	54	41
Severity of disease	5	19	21
Grand total	66	294	58

Length of ICU stay was reported 25 times by 24 studies, which indicates that multiple definitions were used to report on ICU stay. For example, if the ICU stay was reported for all patients and for survivors in one study, these were considered as two different outcomes. Length of stay outcomes, mechanical ventilation outcomes and SOFA were the most popular outcomes. The others were physiological outcomes, and the majority of these were cardiovascular outcomes.

### Potential early phase endpoints for respiratory subgroup in ICU

Based on the National Health Service (NHS) digital data published in 2017 and 2020, approximately 30% of ICU patients will require advanced respiratory support and approximately 80% will require some form of respiratory support,^75–77^ indicating that the respiratory subgroup is one of the most burdened requiring better research and development. Hence a smaller list of 22 outcomes out of the 294 outcomes was chosen, based on the clinical relevance for the subgroup. Table 3 shows the outcome, number of articles (%) reporting the outcome, analysis metric used and aggregation method for these 22 outcomes.

**Table 3.** Potential early phase outcomes, analysis metric, aggregation method for the respiratory subgroup.

Outcome	N^a^ (%)	Analysis metric	Aggregation method	Time point
Duration of mechanical ventilation	20 (34.5%)	Duration of mechanical ventilation – all patients Duration of mechanical ventilation – survivors Duration of mechanical ventilation (invasive and non-invasive) Duration of mechanical ventilation until weaning Days off mechanical ventilation Intubation free days Time before first weaning attempt Time to weaning from mechanical ventilation Weaning duration	Mean ± SD, median (IQR), mean ± SE, median (range), Kaplan–Meier estimate	Extubation, during study period, day 28
Duration of ICU stay	27 (46.6%)	Duration of ICU stay – all patients Duration of ICU stay – survivors only Duration of level 2 ICU stay Duration of level 3 ICU stay	Mean ± SD, median (IQR), Mean ± SE, Median (range)	At ICU discharge, during study period, days 21 and 28
Duration of hospital stay	20 (34.5%)	Duration of hospital stay–all patients Duration of hospital stay–survivors	Mean ± SD, Median (IQR), mean ± SE, median (range)	At hospital discharge, during study period, day 21
ICU-free days	3 (5.2%)	ICU free days	Median (IQR), mean ± SE	28 days
Organ failure–free days	3 (5.2%)	Organ failure free days	n/N, %, n (%)	During study period, 60 days
Ventilator-free days	13 (22.4%)	Ventilator free day	Mean, SD, median (IQR), mean ± SE	21 and 28 days
SOFA score	14 (24.1%)	SOFA (absolute, change, maximum) Non-hepatic SOFA (absolute, change) SOFA corrected (absolute, change)	Mean ± SD, mean (95% CI), median (IQR), mean ± SE	0, 0–6, 8, 0–8 and 9–72 h, baseline, days 0, 1, 3, 5, 7, 10, 14, 21 and 28
MOD score	1 (1.7%)	Absolute value	Text stating ‘no significant difference’	
Lung injury score	1 (1.7%)	Absolute value	Mean ± SD	Baseline, days 2 and 4
Oxygenation index	2 (3.4%)	Absolute value	Mean ± SD, Mean ± SE	Baseline, days 0, 1, 2, 3, 4, 5 and 7
PF ratio	8 (13.8%)	Absolute value, time spend with PaO2: FiO2 < 200 mm Hg	Mean ± SD, Mean ± SE	Baseline, 1, 3, 6, 12, 24, 36 and 48 h, days 0, 1, 2, 3, 4, 5, 6 and 7
Platelets	4 (6.9%)	Absolute value, change score	N, mean, SD, min, max, mean ± SD, median (IQR)	Baseline, 1, 12, 24, 48, 72 and 120 h, days 1, 2 and 6
CRP	1 (1.7%)	Absolute value	Mean ± SD, range	Days 1, 2, 3, 6, 18 and 14
Urine output	1 (1.7%)	Fluid intake, fluid output, fluid balance	Mean ± SD, median (IQR), adjusted mean (95% CI)	Baseline, 8, 0–8 and 9–72 h
Creatinine	4 (6.9%)	Absolute value, change values	Mean, SD, min, max	1, 2, 24, 48, 72 and 120 h
IL-10	4 (6.9%)	Absolute value, change value	Mean ± SD, median (IQR), mean ± SE, range	Baseline, 48–72 h, days 1 and 6
IL-1B	5 (8.6%)	Absolute value, change value	Mean ± SD, mean ± SE, range	0, 8, 0–8 and 9–72 h
IL-6	9 (15.5%)	Absolute value, change value	AUC, mean ± SD, median (IQR), mean ± SE, range	0, 2, 6, 8, 24, 36, 48, 48–72, 72 and 96.5 h, days 1, 2, 3, 4, 6, 7, 8 and 14
IL-8	5 (8.6%)	Absolute value, change value	Mean ± SD, median (IQR), range	0, 8, 24, 48–72 and 96.5 h, days 1, 2 and 4
sRAGE	2 (3.4%)	Absolute value	AUC, mean ± SD, mean ± SE, AUC, sensitivity, specificity	−5, 5 and 30 min, 1, 4 and 6 h, baseline, days 1, 2 and 4
TNFa	7 (12.1%)	Absolute value, change value	Mean ± SD, median, mean ± SE, range	0, 8 and 25 h, baseline, days 1, 2, 4 and 6
CRs static	1 (1.7%)	Absolute value	Mean ± SD	Baseline days 2 and 4

SD: standard deviation; IQR: interquartile range; ICU: Intensive care unit; PF ratio: PaCO_2_/FiO_2_; MOD: Multiple Organ Dysfunction; CRP: C-reative protein; SOFA: sequential organ failure assessment; CI: confidence interval; SE: standard error; AUC: area under the curve.

a Number of articles and percentage (%).

Organ dysfunction outcomes: Duration of mechanical ventilation, organ failure free days and ventilator free days were the organ dysfunction outcomes identified as the potential early phase endpoints. Outcomes related to mechanical ventilation were frequently reported and consisted of the most variable definitions. Mechanical ventilation outcomes definitions included the duration of mechanical ventilation, rate of mechanical ventilation, mechanical ventilation free days, number of ventilated days, time to successful extubation and ventilator free days. Mechanical ventilation outcomes were reported using mean (standard deviation), mean (standard error), median (range), median (inter-quartile range), median (range), Kaplan–Meier estimate and median (95% confidence interval) Kaplan–Meier estimate.

Length of stay outcomes: Length of stay in ICU and hospital and ICU free days were reported using mean (standard deviation), mean (standard error), median (range), median (inter-quartile range), and median (95% confidence interval) Kaplan–Meier estimate. Length of stay outcome definition was reported for all patients and for survivors’ only.

Disease severity scores: Severity scores in Table 3 include SOFA score, MOD score and Lung Injury Score. All three scores are calculated from physiology scores that are routinely collected in the ICU. SOFA was defined in numerous ways such as non-hepatic SOFA and SOFA corrected values which exclude the neurology component. Organ failure was defined using SOFA using different cut-offs such as a change in SOFA > 2 and a SOFA score > 6. SOFA and MOD scores indicate multiple organ failures.

Physiology outcomes: Physiology outcomes were classified into routinely collected data and biomarkers. Looking at the frequency of the outcomes reported, a total of 136/305 (55%) were short-term physiology outcomes, which are routinely collected in the ICUs. Other outcomes are the biomarkers.

**Figure 2.**
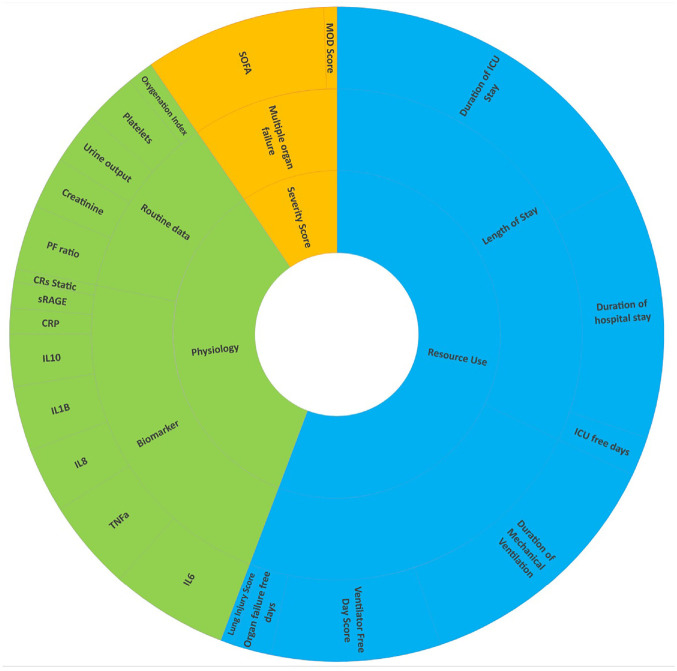
Potential early phase endpoints in respiratory subgroup and classifications (outer circle shows the outcomes, mid-circle represents the subcategory and the inner circle represents the category).

Figure 2 proposes the classification of outcomes that should be reported on an early phase critical care trial. The idea is that all early phase critical care trials report on an organ dysfunction outcome, a length of stay outcome, a disease severity score and physiology outcomes. The physiological outcome should be the one related to the underlying condition. For example, Oxygenation Index and PaO_2_/FiO_2_ ratio (PF ratio) is a physiological outcome associated with ARDS. An early phase study on ARDS patient population should report on the duration of ventilation, length of ICU stay, length of hospital stay, SOFA score, PF ratio and Oxygenation Index. Similarly, a cardiology study may report on a heart dysfunction measure, length of stay, SOFA score, heart rate and a relevant biomarker.

This review did not include COVID studies, and two letters related to COVID studies were identified during article screening. The search on clinicaltrials.gov database for COVID studies identified 191 studies. A total of 2379 outcomes were specified in the database for 191 studies and the number of outcomes per study ranged from 1 to 74. Mortality-related outcomes were specified 227 times, free days scores were specified 306 times and 160 studies specified ventilator free day as an outcome. COVID-19 ordinal clinical progression outcome scale was another outcome frequently specified; however the days and numbers in the scale varied. Other frequently reported outcomes were duration of mechanical ventilation, ICU/hospital length of stay and SOFA.

## Conclusion

This qualitative review looked at surrogate measures and their variability in critical care studies. This review identified about 294 different outcomes. These outcomes were defined and reported clearly within most of the studies, however, their definitions and reporting varied from study to study. Review of trial registration details of the COVID study showed that variability of outcomes and outcome reporting is an issue in COVID studies as well. This makes the comparison between the studies difficult, which shows that there is an urgent need to standardise the outcomes.

Most of the outcomes found in the review can be broadly classified into organ dysfunction outcomes, length of stay outcomes, disease severity scores, routine physiology data and biomarkers. This classification is similar to those proposed in the work of Dodd et al.,^78^ except for adverse events, given the exclusion of outcomes related to adverse events from this review. An estimate of the change in disease severity can be expected to provide an estimate for ‘life impact’ outcomes such as quality of life. A subset of 22 outcomes relevant for the respiratory subgroup was selected for further research. Mechanical ventilation, ventilator free days and organ failure free days indicate organ dysfunction. Mechanical ventilation is a lifesaving intervention in the ICU and ventilation requirements have direct effects on resource use and a direct and attributable effect on mortality. Blackwood et al.^79^ have previously demonstrated the variation in the measurement and reporting of mechanical ventilation outcomes. Furthermore, Contentin et al.^80^ reviewed 128 reports on adult ICU studies and identified 13 different definitions of ventilator free days. The variability in the definition and analysis of mechanical ventilation outcomes was evident in this review as well. The variability in ventilation approaches varies from ICU to ICU and this influences variability even further. Mechanical ventilation outcomes, ventilator free days and organ failure free days indicate organ dysfunction, which increases the risk of death. Organ dysfunction would also impact the length of stay of the patient in the hospital and ICU.

Length of stay outcomes are relevant to clinicians and patients. ICU/hospital length of stay outcomes are clear indicators of resource use. ICU/hospital free days were recommended as endpoints of Phase II studies by the Australia and New Zealand Intensive Care Society (ANZICS) group.^81^ The definition of the length of stay can vary based on whether it is an intervention or medical device study. Intervention studies usually measure the length of stay from the day of randomisation or patient enrolment to the study, while a medical device or an observational study is more likely to measure the length of stay from ICU admission. A point to remember is that a reduction in the duration of mechanical ventilation and length of stay can also be due to patients dying early. Hence combining survivors and non-survivors when reporting the results can be misleading and these outcomes should be reported separately by survival status.

Physiology outcomes were subcategorised into routinely collected outcomes and biomarkers. In Table 3, 13 out of 22 outcomes can be categorised as physiology outcomes. Physiology outcomes such as PF ratio and Oxygenation Index are primary and secondary outcome measures in several ICU studies.^82–85^ However, the magnitude of the association between these variables and mortality is not clear. In the ICU, multiple physiology outcomes can be combined to generate severity scores such as SOFA, which are associated with mortality. Recent developments in data analysis techniques allow the use of these measurements to make predictions.

Many authors have previously reported on the variability of outcome measures in healthcare research. The results of this review once again highlight issues of variability among surrogate outcomes and inconsistency of outcome reporting in critical care studies. Systematic reviews and meta-analyses compare and combine the evidence from various research studies carried out in a field or on an intervention. Heterogeneity in outcome reporting makes a comparison between the studies exceedingly difficult and time-consuming. This is not an effective use of resources and time invested in healthcare research. A Core Outcome Set is the minimum set of outcomes that should be collected and reported. Studies can still collect and report other relevant outcomes and need not be restricted to the outcomes in the core outcome set. The development of a core outcome set and standardisation of these outcomes will reduce the impact of outcome variability.

There are a few limitations to note in this review. First is that the review included all adult ICU studies, except healthy volunteer, end of life and transplant studies. The study was based on the work of the ANZICS group^81^ and was not narrowed down to a specific group of patients, even though conditions requiring ICU care are indiscriminate and heterogeneous. They can cover the entire spectrum of medicine in aetiology from trauma to cardiovascular to psychiatric. These patients often require complex interventions. All these factors might have had an impact on the variability of outcomes. The second limitation is that the study was restricted to those published in the top six journals in critical care over a brief period. Data extraction was limited to the published reports of the studies and protocols were not checked for definitions of outcomes. The impact of this is thought to be minimal because the review was able to identify the different outcomes used in critical care and the variable definitions used which was the purpose of the study. A third limitation was the exclusion of safety outcomes. However, we emphasise that safety reporting is crucial in all phases of clinical studies. A fourth limitation relates to the outcomes being skewed towards respiratory studies. Three of the six high impact journals had a focus on respiratory system, and this could have led to this. The set of outcomes identified can be broadly classified as organ failure outcomes, length of stay outcomes, routinely collected physiology outcome and biomarkers. Similar sets of outcomes can be identified for other patient groups for further testing. A fifth limitation concerns the selection of the articles; all articles from 2010 were included in the review and 10% from other years. The 10% was randomly selected, and this could have potentially excluded a few important outcomes.

## Supplemental Material

sj-pdf-1-ctj-10.1177_17407745231151842 – Supplemental material for Heterogeneity of surrogate outcome measures used in critical care studies: A systematic review Click here for additional data file. Supplemental material, sj-pdf-1-ctj-10.1177_17407745231151842 for Heterogeneity of surrogate outcome measures used in critical care studies: A systematic review by Rejina Verghis, Bronagh Blackwood, Cliona McDowell, Philip Toner, Daniel Hadfield, Anthony C Gordon, Mike Clarke and Daniel McAuley in Clinical Trials
