# Supplementary material for: Heterogeneity of surrogate outcome measures used in critical care studies: A systematic review
Source: Clin Trials. 2023 Mar 22;20(3):307–18. doi: 10.1177/17407745231151842 (PMC10617004; doi:10.1177/17407745231151842)
Supplement: sj-pdf-1-ctj-10.1177_17407745231151842 – Supplemental material for Heterogeneity of surrogate outcome measures used in critical care studies: A systematic review [file sj-pdf-1-ctj-10.1177_17407745231151842.pdf]

Appendix A: Search Strategy

MEDLINE

1. Randomized Controlled Trial/
2. exp Randomized Controlled Trial/
3. randomized controlled trial.mp. [mp=title, abstract, original title, name of substance word, subject heading word, keyword heading word, protocol supplementary concept word, rare disease supplementary concept word, unique identifier]
4. Random Allocation/
5. Double-Blind Method/
6. Clinical Trial/
7. clinical trial.mp. [mp=title, abstract, original title, name of substance word, subject heading word, keyword heading word, protocol supplementary concept word, rare disease supplementary concept word, unique identifier]
8. exp Clinical Trial/
9. (clinical trial, Phase 2 or clinical trial, Phase 3 or controlled clinical trial or randomized controlled trial or multicenter study or clinical trial).pt.
10. (Randomi?ed controlled trial$ or RCT or Random allocation or Randomly allocated or Randomly allocated or (allocated adj2 random)).tw.
11. ((clinical adj trial$) or ((singl$ or doubl$ or treb$ or tripl$) adj (blind$3 or mask$3)) or Single blind or Double blind$ or Placebo$).tw.
12. Placebos/
13. 1 or 2 or 3 or 4 or 5 or 6 or 7 or 8 or 9 or 10 or 11 or 12
14. "american journal of respiratory & critical care medicine".jn.
15. lancet respiratory medicine.jn.
16. chest.jn.
17. critical care medicine.jn.
18. intensive care medicine.jn.
19. 14 or 15 or 16 or 17 or 18
20. Critical Care/
21. Intensive Care Units/
22. Pneumonia/ or Critical Illness/ or Anti-Bacterial Agents/ or Cross Infection/ or Aged/ or Gastric Mucosa/ or Intensive Care Units/ or Middle Aged/ or Critical Care/ or Cefotaxime/
23. (intensive care unit or critical care or critically ill or ICU).tw.
24. Intensive Care Units.mp. [mp=title, abstract, original title, name of substance word, subject heading word, keyword heading word, protocol supplementary concept word, rare disease supplementary concept word, unique identifier]
25. Critical Care.mp. [mp=title, abstract, original title, name of substance word, subject heading word, keyword heading word, protocol supplementary concept word, rare disease supplementary concept word, unique identifier]
26. Critically Ill.mp. [mp=title, abstract, original title, name of substance word, subject heading word, keyword heading word, protocol supplementary concept word, rare disease supplementary concept word, unique identifier]
27. 20 or 21 or 22 or 23 or 24 or 25 or 26
28. 13 and 19 and 27

CINAHL

\# Query Limiters
S50 S37 AND S41 AND S46
S49 S37 AND S42 AND S46
S48 S37 AND S44 AND S46
S47 S37 AND S45 AND S46
S46 S38 OR S39 OR S40
S45 JN Intensive Care Medicine
S44 JN Critical Care Medicine
S43 JN Lancet Respiratory Medicine
S42 JN Chest
S41 JN Critical Care
S40 (MH "Critical Illness") OR (MH "Polyneuropathies") OR (MH "Critically Ill Patients")
S39 (MH "Intensive Care Units")
S38 (MH "Critical Care") OR (MH "Critical Care Nursing")
S37 S26 OR S27 OR S28 OR S29 OR S30 OR S31 OR S32 OR S33 OR S34 OR S35 OR S36
S36 MH "Clinical Trials+"
S35 PT Clinical trial
S34 TX Clinic* n1 trial
S33 TX ((singl* n1 blind*) or (singl* n1 mask*)) or TX ((doubl* n1 blind*) or (doubl* n1 mask*)) or TX ((tripl* n1 blind*) or (tripl* n1 mask*)) or TX ((trebl* n1 blind*) or (trebl* n1 mask*))
S32 TX randomi* control* trial*
S31 MH Random Assignment
S30 TX random* allocat*
S29 TX placebo*
S28 MH Placebos
S27 MH Quantitative Studies
S26 TX allocat* random*
S25 S12 AND S16 AND S21
S24 S12 AND S17 AND S21
S23 S12 AND S19 AND S21
S22 S12 AND S20 AND S21
S21 S13 OR S14 OR S15
S20 JN Intensive Care Medicine
S19 JN Critical Care Medicine
S18 JN Lancet Respiratory Medicine
S17 JN Chest
S16 JN Critical Care
S15 (MH "Critical Illness") OR (MH "Polyneuropathies") OR (MH "Critically Ill Patients")
S14 (MH "Intensive Care Units")
S13 (MH "Critical Care") OR (MH "Critical Care Nursing")
S12 S1 OR S2 OR S3 OR S4 OR S5 OR S6 OR S7 OR S8 OR S9 OR S10 OR S11
S11 MH "Clinical Trials+"
S10 PT Clinical trial
S9 TX Clinic* n1 trial
S8 TX ((singl* n1 blind*) or (singl* n1 mask*)) or TX ((doubl* n1 blind*) or (doubl* n1 mask*)) or TX ((tripl* n1 blind*) or (tripl* n1 mask*)) or TX ((trebl* n1 blind*) or (trebl* n1 mask*))
S7 TX randomi* control* trial*
S6 MH Random Assignment
S5 TX random* allocat*
S4 TX placebo*
S3 MH Placebos
S2 MH Quantitative Studies
S1 TX allocat* random*

EMBASE

1. clinical trial/
2. randomized controlled trial/
3. randomization/
4. single blind procedure/
5. double blind procedure/
6. triple blind procedure/
7. crossover procedure/
8. placebo effect/ or placebo/
9. (((Randomi?ed controlled trial$ or Rct or Random allocation or Randomly allocated or Allocated randomly).tw. or allocated.mp.) adj2 random.tw.) or Single blind$.tw. or Double blind$.tw. or ((treble or triple) adj blind$).tw. or Placebo$.tw. or Case report.tw. [mp=title, abstract, heading word, drug trade name, original title, device manufacturer, drug manufacturer, device trade name, keyword]
10. clinical trial/
11. randomized controlled trial/
12. randomization/
13. single blind procedure/
14. double blind procedure/
15. triple blind procedure/
16. crossover procedure/
17. placebo effect/ or placebo/
18. (((Randomi?ed controlled trial$ or Rct or Random allocation or Randomly allocated or Allocated randomly).tw. or allocated.mp.) adj2 random.tw.) or Single blind$.tw. or Double blind$.tw. or ((treble or triple) adj blind$).tw. or Placebo$.tw. or Case report.tw. [mp=title, abstract, heading word, drug trade name, original title, device manufacturer, drug manufacturer, device trade name, keyword]
19. 1 or 2 or 3 or 4 or 5 or 6 or 7 or 8 or 9 or 10 or 11 or 12 or 13 or 14 or 15 or 16 or 17 or 18
20. Critical Care.jn.
21. "american journal of respiratory and critical care medicine".jn.
22. chest.jn.
23. intensive care medicine.jn.
24. intensive care/
25. intensive care/ or intensive care unit/
26. bacterial infection/ or intensive care unit/ or intensive care/ or pneumonia/
27. critical illness/
28. (Critical Care or critical care unit or intensive care or critical illness).tw.
29. Intensive care.mp. [mp=title, abstract, heading word, drug trade name, original title, device manufacturer, drug manufacturer, device trade name, keyword]
30. intensive care unit/ or patient/ or critically ill patient/ or critical illness/ or intensive care/
31. 24 or 25 or 26 or 27 or 28 or 29 or 30
32. 20 or 21 or 22 or 23
33. 19 and 31 and 32

Appendix B: Systematic Review Outcome Extraction Form

Basic Information

**Full Paper Reference**	
**Design**	
**Patient Group**	
**Aim/Objective**	
**Trial Registration Number**	

Record outcomes and how they were measured (add rows as necessary)

**PRIMARY OUTCOME**	**UNIT OF MEASUREMENT**	**Pre-specified**	**TYPE(Clinical, Surrogate, Composite)**	**Data Collected**	**Reported**	**DEFINITION**
**SECONDARY OUTCOME**	**UNIT OF MEASUREMENT**	**Pre-specified**	**TYPE(Clinical, Surrogate, Composite)**	**Data Collected**	**Reported**	**DEFINITION**

Primary outcome based on the publication
Unit of measurement based on the publication
Pre-specified objective based on the Trial Registration
Categorisation as Surrogate, clinical or composite
Data collected based on the publication
Reported days reported, summary measure used to report (Mean ± SD … etc) the outcome measure

Appendix C: Detailed Table on Outcome Classification

Category<br>Subcategory<br>Outcome
**Biomarker**
**Pulmonary lavage marker**
AFXa levels
Clara cell protein-16
D-dimer
IL-10
IL-1B
IL-6
IL-8
sRAGE
Surfactant protein-D
Thrombin-antithrombin complex
TLR2
TLR4
TNFa
**Systemic marker**
BNP
Inter-cellular adhesion molecule 1
Leukotriene
Procalcitonin
Procalcitonin clearance
Troponin Ic levels
Troponin T
**Blood and Lymphatic System**
**Blood coagulation**
Activated partial thromboplastin time
Antithrombin
Fibrinogen
Partial thrombin time
Prothrombin time
Thrombin-antithrombin complexes
**Full blood count parameter**
Eosinophils
Hematocrit
Hemoglobin
Leucocytes
Lymphocytes
Methemoglobin
Monocytes
Neutrophil
Nitrite

Platelets

Brain

Agitation
- Psychomotor agitation
- RASS
- Time spend agitated

Alcohol Withdrawal
- Clinical Institute Withdrawal Assessment for Alcohol

Analgesic/Sedative use or dose
- Benzodiazepine use
- Fentanyl use
- Lorazepam use
- Midazolam use
- Morphine use
- Opiate use
- Propofol use
- Sedative and Analgesic use
- Sufentanyl use

Antipsychotics use
- Antipsychotics requirement
- Cost for neuroactive drugs
- Haloperidol use

CNS parameter
- Cerebral spinal fluid concentration
- Encephalopathy score

Cognitive score
- Adapted Cognitive Exam score

Coma
- Coma free days
- Duration in coma stage

Delirium
- Coma and delirium free days
- Delirium free days
- Duration of delirium
- Incidence of delirium

Epilepsy
- Phenobarbital use

Paralytic agent
- Paralytic agents

Sedation
- Accurately sedated
- Cost for planned sedatives
- Cost for unplanned sedatives
- Sedation
- Time spent sedated

Stress

Post-traumatic stress

CVS

Biochemistry

Arterial base excess
Base deficit
Lactate dehydrogenase

Hemodynamic parameter

Blood pressure
Cardiac index
Central venous pressure
Diastolic arterial pressure
Diastolic Blood Pressure
Global ejection fraction
Global end diastolic volume index
Heart Rate
Left ventricular stroke work index
Mean arterial pressure
Pulmonary Arterial Occlusion Pressure
Pulmonary artery wedge pressure
Right Arterial Pressure
Stroke volume
Stroke volume index
Systolic arterial pressure
Systolic blood pressure
Time to clinical stabilization

Macrovascular parameter

Intravascular volume expansion

Microcirculatory parameter

De Backer score
Heterogeneity index
Microvascular flow index
Perfused vessel
Perfused vessel density
Proportion of perfused vessel
Systemic vascular resistance index
Total vessel density

Oxygenation Parameters

Cutaneous tissue oxygen partial pressure
Mixed venous oxygen saturation
Oxygen consumption
Oxygen delivery
Oxygen delivery index
Oxygen extraction
Oxygen Extraction Ratio

ScvO2
VO2
VO2I

Vasoactive drug use

Catecholamine use
Dopamine use
Epinephrine use
Inotropes use
Milrinone use
Norepinephrine use
Vasoactive drug use
Vasodilator use
Vasopressor free days
Vasopressor use

GI/Liver

Biochemistry

Albumin
ALT
AST
Bilirubin
GGT

Infection

Antimicrobial use

Antibiotic days
Antibiotic free days
Antibiotic use
Antimicrobial therapy
Antipsychotics requirement
Corticosteroids use
Duration of antibiotic exposure
Duration of antibiotic therapy

Bacterial Infection

Antibiotic-associated infections
Antibiotic-resistant pathogens
Bacteraemia
MDR pathogen infection

Blood stream infection

Blood culture infection
Catheter-related bloodstream infection

Catheter-related infection

Catheter infection
Catheter-related infection

CNS infection

CSF infection

Colonization

- Catheter colonization
- Endotracheal colonization
- Oropharyngeal colonization
- Probability of remaining colonization-free

General ICU acquired infection

- Duration acquired infection
- ICU acquired infection
- Infection
- Microbiological eradication
- Microbiological safety
- Other nosocomial infection
- Rate of all ICU-acquired infections
- Rate of ICU-acquired infections

Hematology

- CRP
- WBC

Respiratory infection

- Duration acquired pneumonia
- MRSA Pneumonia
- Nosocomial Pneumonia
- Pneumonia
- Poly microbial Pneumonia
- Positive respiratory culture
- Respiratory infection
- VAP
- Ventilator associated tracheobronchitis

Sequential nosocomial infection

- Nosocomial infection

Surgical infection

- Surgical site infection
- Wound infection

Urinary Infection

- UTI

Vital Sign

- Temperature

Kidney

Acute renal failure

- Acute kidney injury
- Acute renal failure
- Catheter dyfunction
- Continuous Veno-Venous Hemofiltration
- Dialysis Sessions Delivered
- Down-time per patient-day
- Hemodialysis
- Renal Failure free days

Renal Replacement Therapy

Biochemistry

Creatinine

eGFR

Potassium

Urea

Dialysis

Functional circuit life

Renal Replacement Therapy

RRT free days

Transfusion

Fluid

Blood product use

Colloid

Fluid

Urine output

Metabolic

Biochemistry

Glucagon

Glucose

Glycemia

Fats

Arachidonic acid

Docosahexaenoic Acid

Eicosapentaenoic Acid

Fatty acid binding protein

Lipid intake

Prostaglandin

Insulin

Insulin

Metabolic parameter

Energy intake

Gastric Emptying Coefficient

Glycemia

Mean efficacious volume

Muscle Strength

Muscle Strength

Protein

Amino acid intake

Plasma C-peptide

Prolactin plasma levels

Protein

Resource

Additional intervention

Additional surgery

Rescue therapies

Hospital Stay

Cost of hospitalization

Duration of Hospital stay

Hospital discharge

Hospital-free days

ICU Stay

Duration of ICU stay

ICU free days

ICU re-admission

Nursing intensity

Nursing intensity

Rehabilitation

Walk test

Respiratory

Airway

Endotracheal intubation

Failed extubation

Intubation

Need for intubation

Reason for intubation

Re-intubation

Self extubation

Time to first extubation

Time to succesful extubation

Tracheostomy

Time to intubation

ARDs development

ALI/ARDS

Lung injury Score

Lung Compliance

CRs static

Peak inspiratory pressure

Peak Pressure

Lung function

FEV1

Forced vital capacity

Total lung capacity

Lung perfusion

Ventilation–perfusion ratio

Mechanical Ventilation

Duration of combined CMV mode

Duration of mechanical ventilation

Duration of non-invasive ventilation

Duration of weaning

Manual settings per patient
Pressure support ventilation
Rate of mechanical ventilation
Rate of non-invasive ventilation
Ventilator free days
Weaning duration
Weaning success rate

Non-invasive ventilation

Duration of non-invasive ventilation
Need for non-invasive ventilation

Oxygenation

FiO2
Fraction of inspired oxygen
Oxygenation Index
PaO2
PEEP
PF Ratio
Respiratory Rate
SaO2

Pulmonary oedema

Pulmonary oedema

Thoracostomy parameter

Chest tube drainage

Ventilation parameter

Accessory respiratory muscle
Arterial blood gases
Arterial pH
Bicarbonate
Exhaled CO2
Minute ventilation
Minute volume
PaCO2
pH
Pressure Support
Respiratory Rate
Tidal volume
Tidal Volume per Ideal Body Weight

Severity of Disease

Acute Physiology and Chronic Severity Score

APACHE

Organ failure

Acute respiratory failure
Cardiovascular failure
Central nervous system failure
ECMO

- Extrapulmonary organ failures
- Hematologic failure
- Hepatic failure
- Kelly Score
- MOD Score
- Organ failure
- Organ failure free days
- Pneumothorax
- Renal failure
- SOFA

Pancreatic Inflammation

- Modified CT score

Pain score

- VAS Score

Trauma severity score

- Glasgow outcome scale score
- Injury severity score
